# Identification of a 9‐gene prognostic signature for breast cancer

**DOI:** 10.1002/cam4.3523

**Published:** 2020-10-14

**Authors:** Zelin Tian, Jianing Tang, Xing Liao, Qian Yang, Yumin Wu, Gaosong Wu

**Affiliations:** ^1^ Department of Thyroid and Breast Surgery Zhongnan Hospital of Wuhan University Wuhan China

**Keywords:** breast cancer, gene expression omnibus, nomogram, prognostic signature

## Abstract

Breast cancer (BRCA) is the most common cancer among women and is the second leading cause of cancer death in women. In this study, we developed a 9‐gene prognostic signature to predict the prognosis of patients with BRCA. GSE20685, GSE42568, GSE20711, and GSE88770 were used as training sets. The Kaplan–Meier plot was constructed to assess survival differences and log‐rank test was performed to evaluate the statistical significance. The overall survival (OS) of patients in the low‐risk group was significantly higher than that in the high‐risk group. ROC analysis indicated that this 9‐gene signature shows good diagnostic efficiency both in OS and disease‐free survival (DFS). The 9‐gene signature was further validated through GSE16446, GSE7390, and TCGA‐BRCA datasets. We also established a nomogram that integrates clinicopathological features and 9‐gene signature. The analysis of the calibration plot showed that the nomogram has good prognostic performance. More convincingly, real‐time reverse transcription‐polymerase chain reaction (RT‐PCR) results indicated that the protective prognostic factors in BRCA patients were downregulated, whereas the dangerous prognostic factors were upregulated. The innovation of this article is not only constructing a prognostic gene signature, but also combining with clinical information to further establish a nomogram to better predict the survival probability of patients. It is worth mentioning that this signature also does not depend on other clinical factors or variables.

## Graphical abstract

This study developed a novel 9‐gene signature prediction model to predicting the prognosis of BRCA patients. The results show that the prediction model is a powerful predictor of OS and DFS. The establishment of this model may help BRCA patients to formulate more accurate treatment plans and improve the prognosis of BRCA.

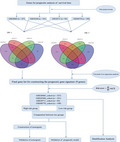

## 1 INTRODUCTION

BRCA is the most common cancer in women. The 2018 GLOBOCAN report disclosed that approximately 2.1 million women worldwide were diagnosed with BRCA in 2018, accounting for one‐fourth of all cancer cases among women.^1^ According to clinicopathological criteria, BRCA is divided into four subtypes Luminal A, Luminal B, Erb‐B2 overexpression, and Basal‐like, represents a convenient approximation.^2^ Ihemelandu study indicated that Luminal A type accounts for 50% of BRCA patients, Luminal B, Erb‐B2 overexpression, and Basal‐like subtypes accounted for 14.1%, 12.7%, and 23.2%, respectively.^3^ For ER receptor‐positive BRCA, tamoxifen, and aromatase inhibitors are effective endocrine therapies.^4^ For HER2‐positive BRCA, the monoclonal antibody trastuzumab and lapatinib have been approved as the most specific molecular targeting drugs.^5^, ^6^ With the development of medical technology, the prognosis of BRCA has been significantly improved. However, the prognosis of advanced BRCA is still not optimistic.^7^

In recent years, detailed information on prognostic assessment of cancer patients has been available via microarray analysis and whole‐genome sequencing.^8^, ^9^ Genomics and molecular characteristics research have significantly improved the biological understanding of BRCA, elucidated the inherent molecular subtypes and genetic driving mechanisms of BRCA.^9^, ^10^ Molecular diagnosis can help to determine whether chemotherapy is needed after surgery and predict the corresponding risk of distant metastasis.^11^ Many studies have demonstrated the prognostic role of gene expression characteristics based on gene expression arrays.^12^, ^13^, ^14^ The mainstream commercial panels for BRCA predictive markers mainly include: Oncotype DX (21 genes assay), MammaPrint (70 gene assay), Prosigna (50 genes assay), Breast cancer index (7 gene assay), and EndoPredict (11 gene assay).^11^, ^15^, ^16^ Molecular diagnosis has received increasing attention as a potential non‐invasive monitoring option for the risk of recurrence in BRCA patients. In this study, we developed a 9‐gene prognostic signature, providing hope for more personalized treatment interventions for BRCA patients.

## 2 MATERIALS AND METHODS

### 2.1 Data processing

**FIGURE 1:**
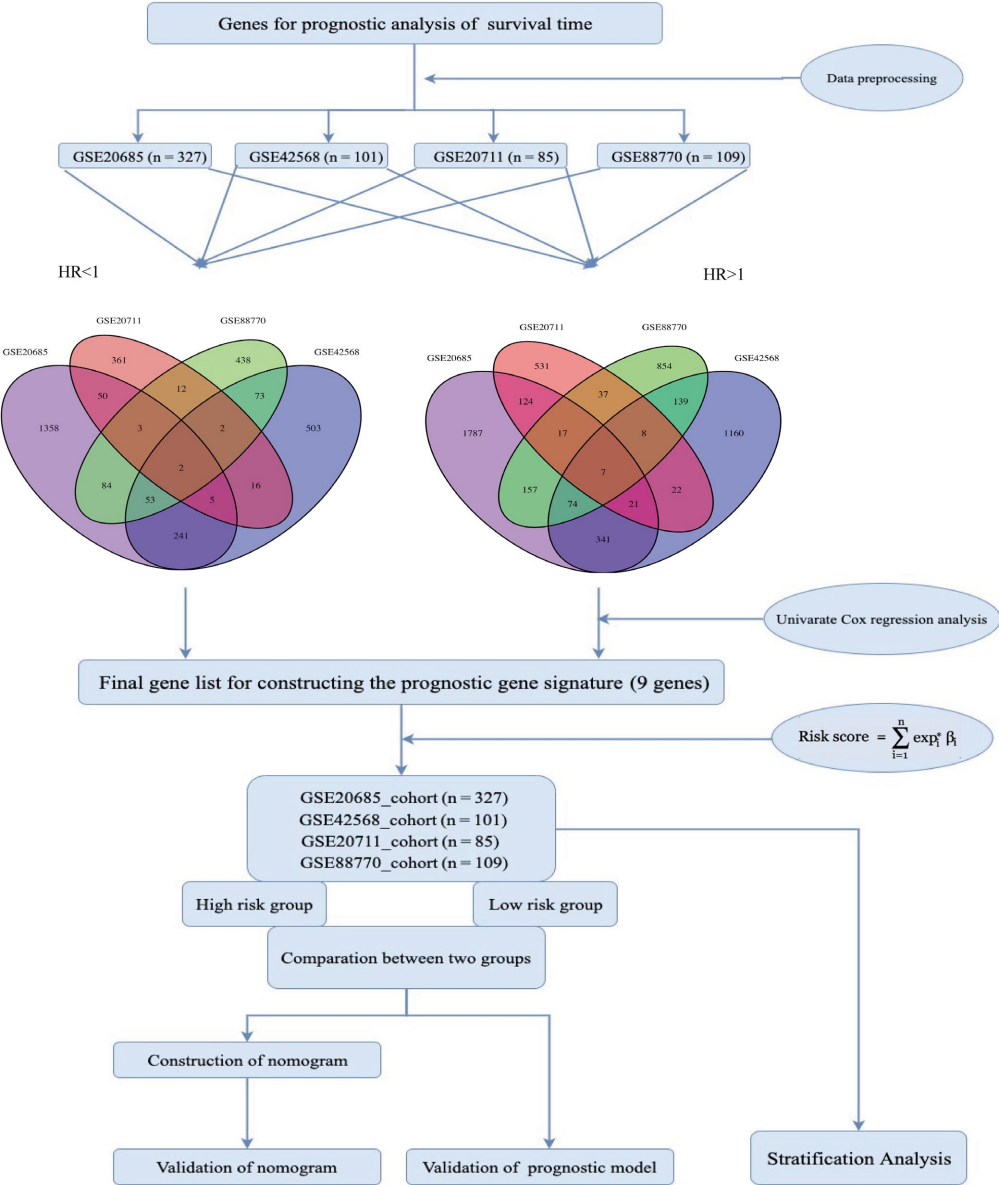
Flow diagram of date preparation, processing, analysis, and validation

The workflow of data acquisition, pre‐processing, gene signature generation, and verification is represented in Figure 1. The original gene expression data and clinical information were obtained from the GEO and TCGA databases. We downloaded the original expression profile and used the robust multi‐array average algorithm to perform background correction and quantile normalization. The ComBat method was used to remove batch effects. We also downloaded the FPKM‐standardized RNA‐seq data and the clinical information from the BRCA cohort in TCGA database. After removing incomplete clinical information and cases of normal samples, a total of 622 cases were included in training sets (327 cases in GSE20685 cohort, 101 cases in GSE42568 cohort, 85 cases in GSE20711 cohort, and 109 cases in GSE88770 cohort) and 1408 cases were included in validation sets (120 cases in GSE16446 cohort, 198 cases in GSE7390 cohort, 1090 cases in TCGA‐BRCA cohort).

### 2.2 Prognostic signature construction and validation

A univariate Cox proportional hazard regression model was developed to screen genes associated with prognosis in each training dataset. In a Cox proportional hazards model, a hazard ratio greater than one indicates that the event hazard increases, and thus the length of survival decreases. In simple terms: HR = 1 ‐ No effect; a HR < 1 ‐ Reduction in the hazard; HR > 1 ‐ Increase in Hazard. In cancer studies, a covariate with HR > 1 is called a dangerous prognostic factor, and a covariate with HR < 1 is called a protective prognostic factor. Hazard ratios |HR| > 1 and *p*‐value < 0.05 (*p*‐value < 0.05 in GSE42568, GSE20711, and GSE88770; *p*‐value < 0.01 in GSE20685) were used to screen candidate genes related to OS from each dataset. To improve reliability, only the common genes in the four datasets were used as prognostic gene signature (*STXBP3*,*PKN2*, *TCAP*,*STARD3*,*CDR2L*,*PNMT*,*GPR4*,*ANGPT2*, and *CAPN5*). The patients were classified as high‐ or low‐risk group according to the optimum cut‐off risk score. The risk score formula is as follows:

$$\mathrm{Risk}\mathrm{score}=\sum_{i=1}^{n}\exp_{i}^{*}\beta_{i}$$

where *n* represents the number of prognostic genes, exp*_i_* is the expression value of gene *i*, and *β_i_* is the univariate Cox regression coefficient of gene *i* (*i* = 9). We used GSE20685, GSE42568, GSE20711, and GSE88770 as training sets; GSE16446, GSE7390, and TCGA‐BRCA as external validation sets to validate the accuracy of the 9‐gene prognostic signature. The cut‐off value for distinguishing high‐risk and low‐risk patients in the validation sets were determined by calculating the optimal cut‐off regarding the 9‐gene signature.

### 2.3 Construction and verification of the nomogram

In this study, an “rms” R package was used to generate the nomogram containing clinical information and 9‐gene signature. The C index and calibration plot were used to evaluate the accuracy of nomogram. The prediction efficiency of the nomogram is shown in the calibration plot, where the 45° dotted line indicates the best prediction.

### 2.4 Acquisition of human BRCA samples

BRCA and paired adjacent samples were collected from patients who underwent breast surgery at Zhongnan Hospital of Wuhan University. All samples were obtained with informed consent of patients. The tissues were immediately stored in liquid nitrogen for further experiments. The Ethics Committee of Zhongnan Hospital of Wuhan University approved the use of these samples for total RNA isolation and RT‐PCR analysis. The inclusion criteria: (a) Patients with BRCA; (b) Patients with clear clinical information (ERα, PR, HER2, Lymph node metastasis, Pathological grade). The exclusion criteria: (a) Patients without complete clinical information; (b) Patients without endocrine or radiotherapy before surgery; (c) Patients without other concomitant diseases. This study included 40 pairs of samples and the clinical information of the patients is shown in Table S1.

### 2.5 Total RNA extraction and QPCR analysis

RNeasy plus mini kits (74134, Qiagen) was used to extract total RNA from frozen tissues according to the manufacturer's protocol. The concentration of RNA was determined by spectrophotometry (NanoDrop, Thermo Scientific) and A_260_/A_280_ ratio was measured to ensure RNA purity. Two μg of total RNA was reverse‐transcribed to complementary DNA (cDNA) via the HiScript II Q RT SuperMix (Vazyme) according to the manufacturer. RT‐PCR was performed using 10 μl of the 2 × SYBR Master Mix (TOYOBO) and 2 μl cDNA (50 ng/μl) with 1 μl each of the forward and reverse primers (10 μmol/L), add nuclease‐free water to the final volume of 20 μL. RT‐PCR was conducted in triplicate. GAPDH was used as internal control, and the 2^−ΔΔCt^ values were normalized to its levels. The primer sequences for RT‐PCR used in this study are shown in Table S2.

### 2.6 Gene set enrichment analysis

According to the optimum cut‐off risk score, BRCA samples were divided into high‐risk and low‐risk groups. We used the GSEA software (GSEA version 4.0.3) to perform a gene set enrichment analysis (GSEA) between high‐ and low‐ risk groups. The c2.cp.kegg.v6.2.symbols.gmt gene set was selected as the reference gene set. The most significant first 5 Kyoto Encyclopedia of Genes and Genomes (KEGG) pathways were screened (FDR <25%).

### 2.7 Statistical analysis

In this study, we used Cox proportional hazards regression analysis to construct the prediction model, which is more useful than Kaplan–Meier curves and log‐rank tests since it works for both quantitative predictor variables and for categorical variables, and allows to assess simultaneously the effect of several risk factors on survival time. The Kaplan–Meier method was used to evaluate the differences in OS and DFS in patients with low‐risk and high‐risk group, and log‐rank tests were used to evaluate the statistical significance of the differences between groups. Multivariate Cox regression analysis and stratification analysis were used to assess whether the 9‐gene signature was independent of other clinical characteristics. The “survivalROC” R package was used for time‐dependent receiver operating characteristic (ROC) analysis, and the prognostic performance was verified by comparing the area under the ROC curve (AUC). *p* < 0.05 was considered statistically significant. All statistical tests were performed by R software (version 3.6.1).

## 3 RESULTS

### 3.1 Prognostic signature generation

In order to identify candidate prognostic genes that are significantly associated with OS, we performed univariate Cox proportional hazard regression analysis on each data. Using *p* < 0.05 and HR < 1 as the cutoff criteria, 1797 genes in GSE26085, 895 genes in GSE42568, 450 genes in GSE20711, and 666 genes in GSE88770 were identified as candidate protective prognostic factors. Using *p* < 0.05 and HR > 1 as the cutoff criteria, there were 2528 genes in GSE26085, 1771 genes in GSE42568, 766 genes in GSE20711, and 1292 genes in GSE88770 were identified as candidate dangerous prognostic factors. The common genes in four datasets were retained as prognostic genes. Two protective prognostic factors (*STXBP3*, *PKN2*) and seven dangerous prognostic factors (*TCAP*,*STARD3*,*CDR2L*,*PNMT*,*GPR4*,*ANGPT2*,*and CAPN5*) were finally obtained. The general information for these genes is indicated in Table S3. The prognostic correlations of the 9‐genes in each dataset associate with OS are shown in Table 1 (HR value, 95% confidence interval, *p* value).

**TABLE 1:** Univariate regression analysis was performed on the OS of 9 genes and BRCA patients in training sets

Genes	GSE26085		GSE42568		GSE20711		GSE88770	
	HR(95%CI)	*p*‐value	HR(95%CI)	*p*‐value	HR(95%CI)	*p*‐value	HR(95%CI)	*p*‐value
STXBP3	0.27 (0.12‐0.60)	1.46E−03	0.17 (0.08‐0.37)	4.46E−06	0.12 (0.03‐0.45)	1.91E−03	0.12 (0.03‐0.51)	4.01E−03
PKN2	0.58 (0.34‐0.99)	4.86E−02	0.31 (0.14‐0.69)	3.83E−03	0.24 (0.07‐0.88)	3.08E−02	0.16 (0.03‐0.95)	4.38E−02
TCAP	1.69 (1.25‐2.28)	5.91E−04	1.86 (1.16‐2.97)	9.81E−03	2.38 (1.35‐4.19)	2.74E−03	6.87 (1.61‐29.21)	9.08E−03
STARD3	1.33 (1.12‐1.58)	1.36E−03	1.64 (1.20‐2.25)	1.96E−03	1.35 (1.04‐1.74)	2.31E−02	1.62 (1.01‐2.60)	4.69E−02
CDR2L	1.66 (1.21‐2.29)	1.91E−03	2.09 (1.32‐3.32)	1.74E−03	2.02 (1.13‐3.63)	1.79E−02	5.89 (2.21‐15.70)	3.85E−04
PNMT	1.20 (1.04‐1.38)	1.26E−02	1.35 (1.09‐1.67)	5.41E−03	1.44 (1.21‐1.72)	4.71E−05	2.01 (1.23‐3.29)	5.26E−03
GPR4	2.46 (1.15‐5.26)	2.02E−02	7.63 (2.85‐20.39)	5.14E−05	13.26 (2.21‐79.45)	4.65E−03	6.03 (1.80‐20.26)	3.65E−03
ANGPT2	1.56 (1.07‐2.26)	2.08E−02	4.05 (1.60‐0.25)	3.09E−03	2.41 (1.21‐4.81)	1.28E−02	2.70 (1.13‐6.46)	2.54E−02
CAPN5	1.69 (1.08‐2.63)	2.11E−02	6.00 (2.20‐16.41)	4.78E−04	4.50 (2.12‐9.55)	9.16E−05	2.49 (1.09‐5.69)	3.13E−02

### 3.2 Analysis of the prognostic signature

**FIGURE 2:**
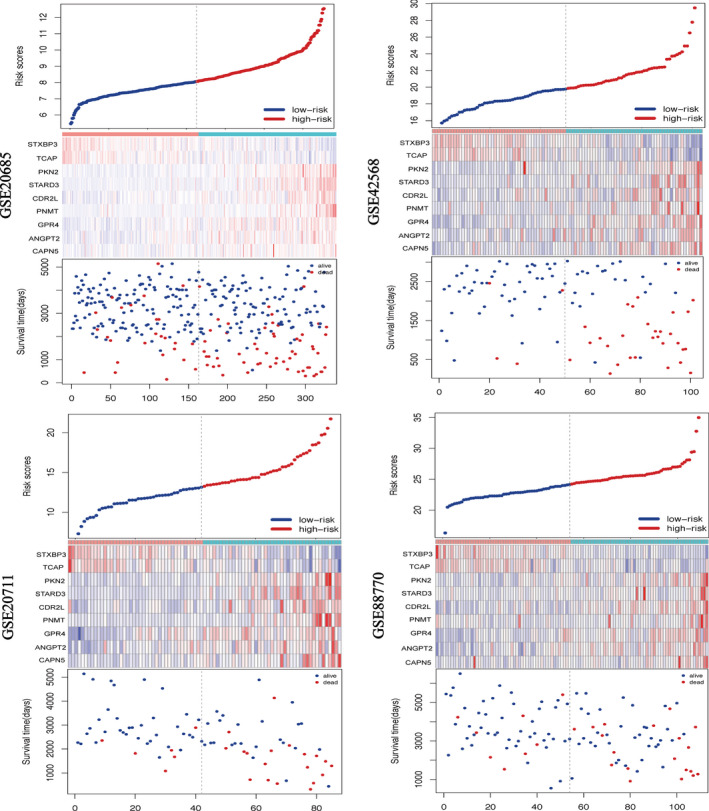
Analysis of risk score for BRCA patients in four datasets. In each graph, from top to bottom were risk score distribution, gene expression profile, and patient survival status. The black dashed line represents the median value of the risk score, which was used as a boundary to divide patients into high‐risk and low‐risk groups

**FIGURE 3:**
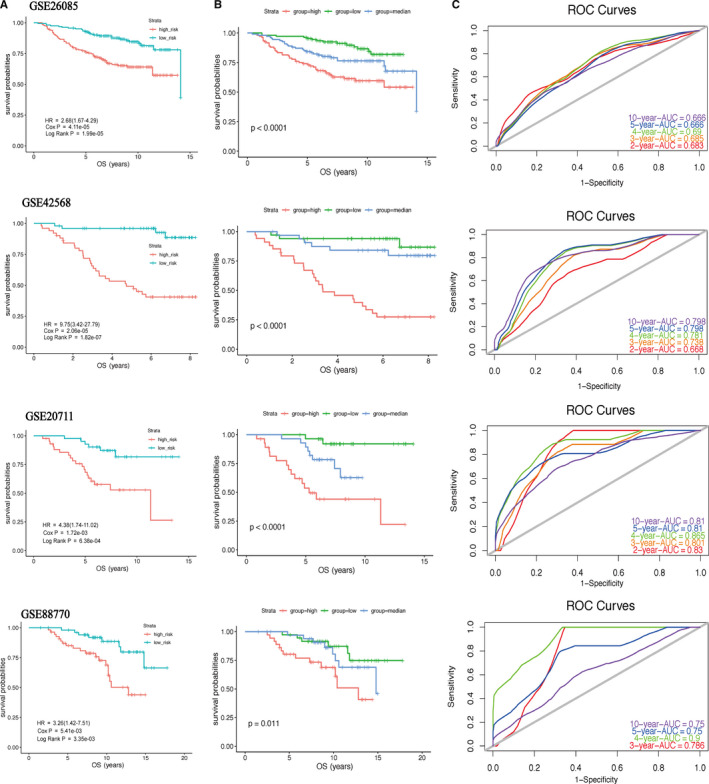
Kaplan–Meier plot (A, B) and ROC curves (C) for 9‐gene signature in four datasets. The OS of patients in the high‐risk group was lower than that in the low‐risk group. *p* value < 0.05 (log‐rank test) was considered statistically significant

**FIGURE 4:**
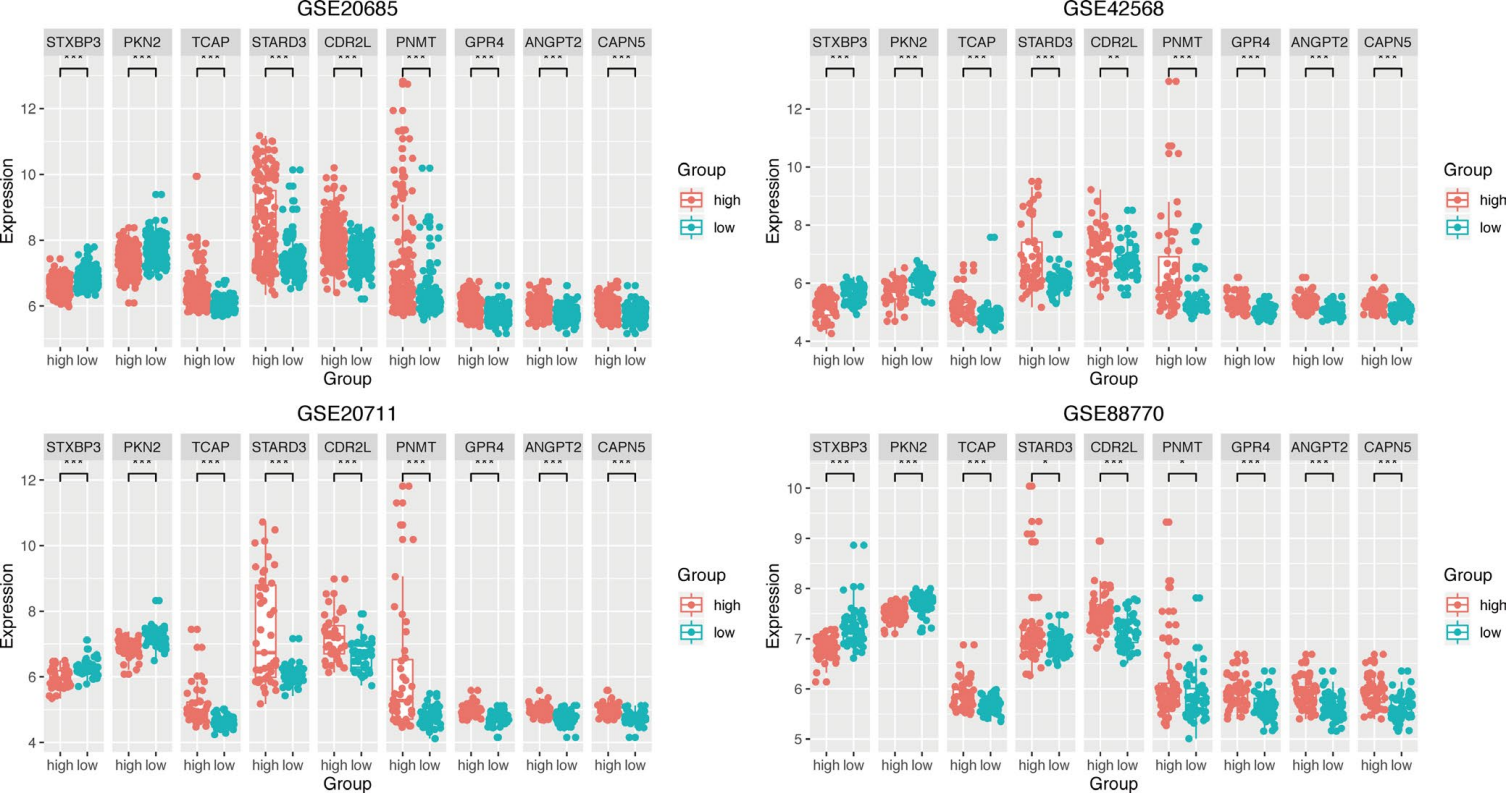
Box plot visualization of gene expression levels of 9‐gene signature in training sets. Patients in the high‐risk group had higher expression of dangerous prognostic factors, whereas in the low‐risk group had lower expression of protective prognostic factors. *p* value < 0.05 (*t* test) was considered statistically significant. *p < 0.05; **p < 0.01; ***p < 0.001; ****p < 0.0001

In Figure 2, the ranking was based on the risk score values of 9‐gene signatures from low to high, the risk score distribution, risk gene expression, and patient survival status of GSE20685, GSE42568, GSE20711, and GSE88770 datasets were shown, respectively. Kaplan–Meier plot indicated that the low‐risk group patients has a better prognosis (GSE20685: HR = 2.68(1.67‐4.29), *p* value = 1.99e‐05; GSE42568: HR = 9.75(3.42‐27.79), *p* value = 1.82e‐07; GSE20711: HR = 4.38(1.74‐11.02), *p* value = 6.38e‐04; GSE88770: HR = 3.26(1.42‐7.51), *p* value = 3.35e‐03) (Figure 3A). For further verification, we divided patients into three groups: high‐, medium‐, and low‐risk based on the value of the risk score. The results also proved that the higher the risk score, the worse the patient's OS (Figure 3B). The ROC curves based on 9‐gene signature showed that, as time went on, the AUC values of the four datasets have remained at a relatively satisfactory value, which can effectively predict OS (Figure 3C). Figure 4 indicates the expression levels of these 9 prognostic genes between the low‐risk and high‐risk groups. The results indicated that high‐risk group patients had higher dangerous prognostic factor expression, while low‐risk group patients had lower protective prognostic factor expression.

### 3.3 9‐gene prognostic signature is independent of other clinicopathological factors

Multiple Cox regression analysis was used to assess whether the 9‐gene signature could be used as an independent prognostic factor. The results indicated that in four independent datasets, the 9‐gene signature can be used as an independent prognostic factor, and its predictive ability is independent of other clinicopathological factors (GSE20685: HR = 2.234(1.3668‐3.651), *p* value = 0.001; GSE42568: HR = 8.388(2.908‐24.196), *p* value = 8.33e‐05; GSE20711: HR = 3.857(1.522‐9.772), *p* value = 0.012; GSE88770: HR = 2.860(1.239‐6.600), *p* value = 0.014) (Table 2). In addition, lymph node metastasis status can also be used as an independent prognostic factor.

**TABLE 2:** Univariate and multivariate Cox regression analyses were performed on the gene signatures and OS of BRCA patients in training sets

Variables		Patients(N)	Univariate analysis		Multivariate analysis	
			HR(95% CI)	*p*	HR(95% CI)	*p*
GSE20685						
T_stage	I/II	101/188	1.136(0.664‐1.944)	0.642	0.732(0.4150‐1.292)	0.281
T_stage	I/III	101/38	4.663(2.550‐8.526)	**5.7e−07**	1.824(0.889‐3.744)	0.101
M_stage	M0/M1	319/8	5.204(2.391‐11.33)	**3.22e−05**	1.475(0.6029‐3.609)	0.394
Nodes	−/+	137/190	3.785(2.163‐6.623)	**3.12E−06**	3.391(1.869‐6.155)	**5.91e−05**
Risk score	Low/High	163/164	2.678(1.672‐4.287)	**4.11e−05**	2.234(1.3668‐3.651)	**0.001**
GSE42568						
T_stage	I/II	34/67	2.311(1.002‐5.332)	**0.049**	1.438(0.613‐3.374)	0.404
Nodes	−/+	44/57	4.556(1.877‐11.050)	**0.001**	3.601(1.460‐8.882)	**0.005**
Grade	I/II	10/40	1.857(0.232‐14.86)	0.559		
Grade	I/III	10/51	6.208(0.839‐45.96)	0.073		
ER	−/+	34/67	0.532(0.2679‐0.086)	**0.041**	0.461(0.230‐0.922)	**0.028**
Risk score	Low/High	50/51	9.746(3.418‐27.790)	**2.06e−05**	8.388(2.908‐24.196)	**8.33e−05**
GSE20711						
T_stage	I/II	46/39	1.833(0.838‐4.044)	0.133		
Nodes	−/+	29/56	3.115(1.067‐9.095)	**0.037**	2.483(1.082‐7.322)	**0.046**
Grade	I/II	13/4	1.277 (0.414‐14.300)	0.843		
Grade	I/III	13/68	2.433(0.571‐10.360)	0.229		
ER	−/+	43/42	0.557(0.245‐1.265)	0.162		
HER2	−/+	61/24	2.533(1.146‐5.597)	**0.021**	1.412(0.606‐3.290)	0.424
Risk score	Low/High	42/43	4.378(1.739‐11.020)	**0.001**	3.857(1.522‐9.772)	**0.012**
GSE88770						
Nodes	−/+	62/47	2.455(1.100‐5.476)	**0.028**	2.338(1.035‐5.280)	**0.041**
Grade	I/II	13/90	2.190(0.513‐9.338)	0.290		
ER	−/+	11/98	0.997(0.338‐2.937)	0.996		
Ki67(%)	~15/15 ~ 30	71/23	3.565(1.525‐8.333)	**0.003**	3.160(1.344‐7.429)	**0.008**
Ki67(%)	~15/30~	71/15	1.827(0.642‐5.200)	0.259	1.515(0.527‐4.358)	0.441
Risk score	Low/High	54/55	3.263(1.418‐7.509)	**0.005**	2.860(1.239‐6.600)	**0.014**

### 3.4 Nomogram development and validation

**FIGURE 5:**
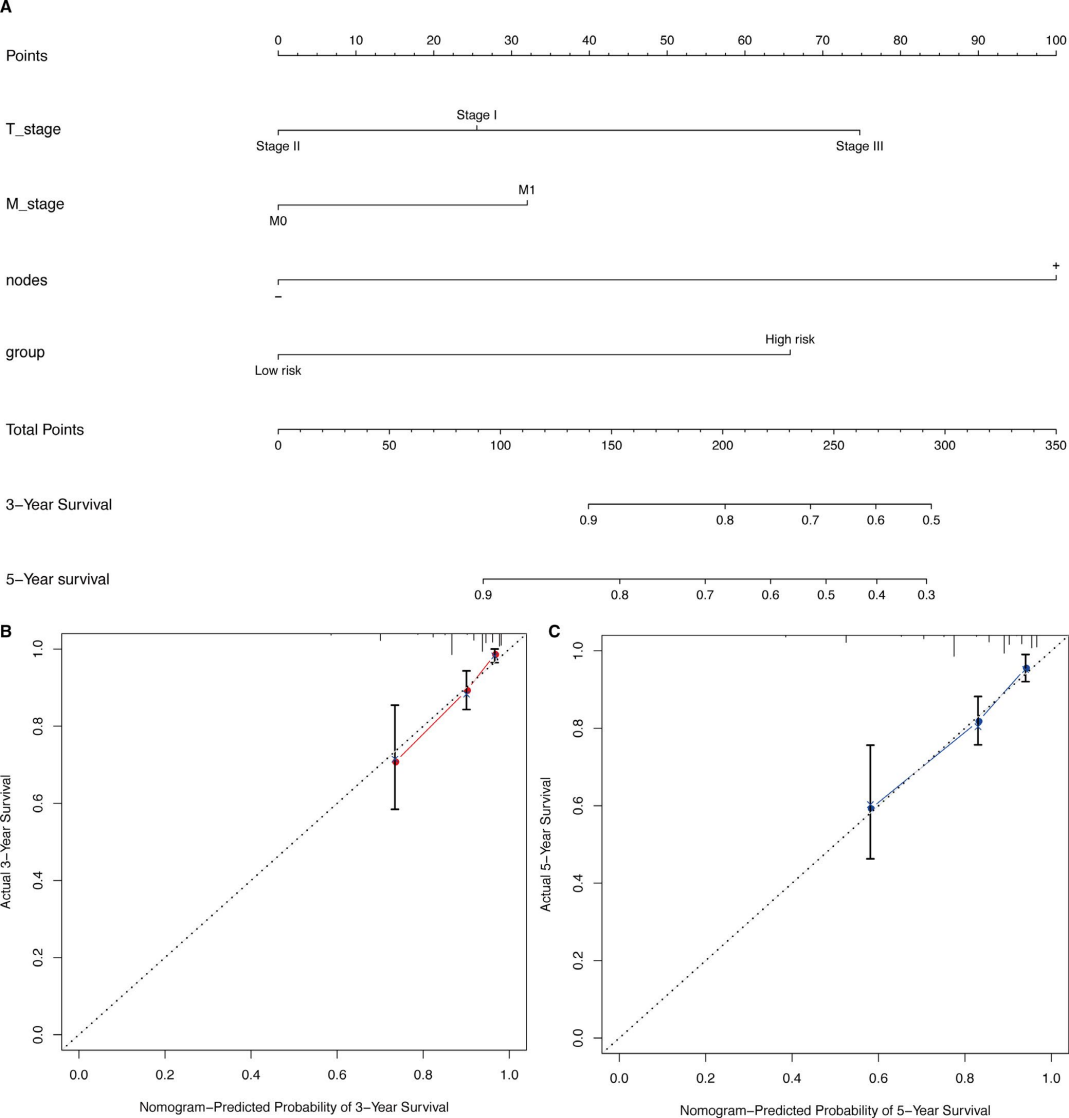
Nomogram of the GSE20685 datasets was used to predict the OS of BRCA patients. To use the nomogram, each variable axis contains a values that should be matched to the each individual patient with a line upward to determine the number of points received for each variable value. The sum of these numbers is located on the total points axis, and a line should be drawn downward to the survival axis to determine the probability of OS (A). The 3‐year and 5‐year OS calibration plot showed that the nomogram has good prediction accuracy (B, C)

Nomogram plot the 9‐gene signature and clinicopathological factors on the same plane, and integrates them via proportional line segments to indicate the relationship between variables in the prediction model. In four independent datasets, we constructed a nomogram to better quantitatively predict the three‐year and five‐year survival rate (Figure 5A and Figure S1A‐C). The calibration curve (Figure 5B, C and Figure S1D‐G) and C index (GSE20685: Concordance=0.735 (se=0.028); GSE42568: Concordance = 0.828(*SE* = 0.034); GSE20711: Concordance = 0.726(*SE* = 0.047); GSE88770: Concordance = 0.76(*SE* = 0.055)) indicated that the prediction results of the nomogram have good accuracy.

### 3.5 Stratification analysis

**FIGURE 6:**
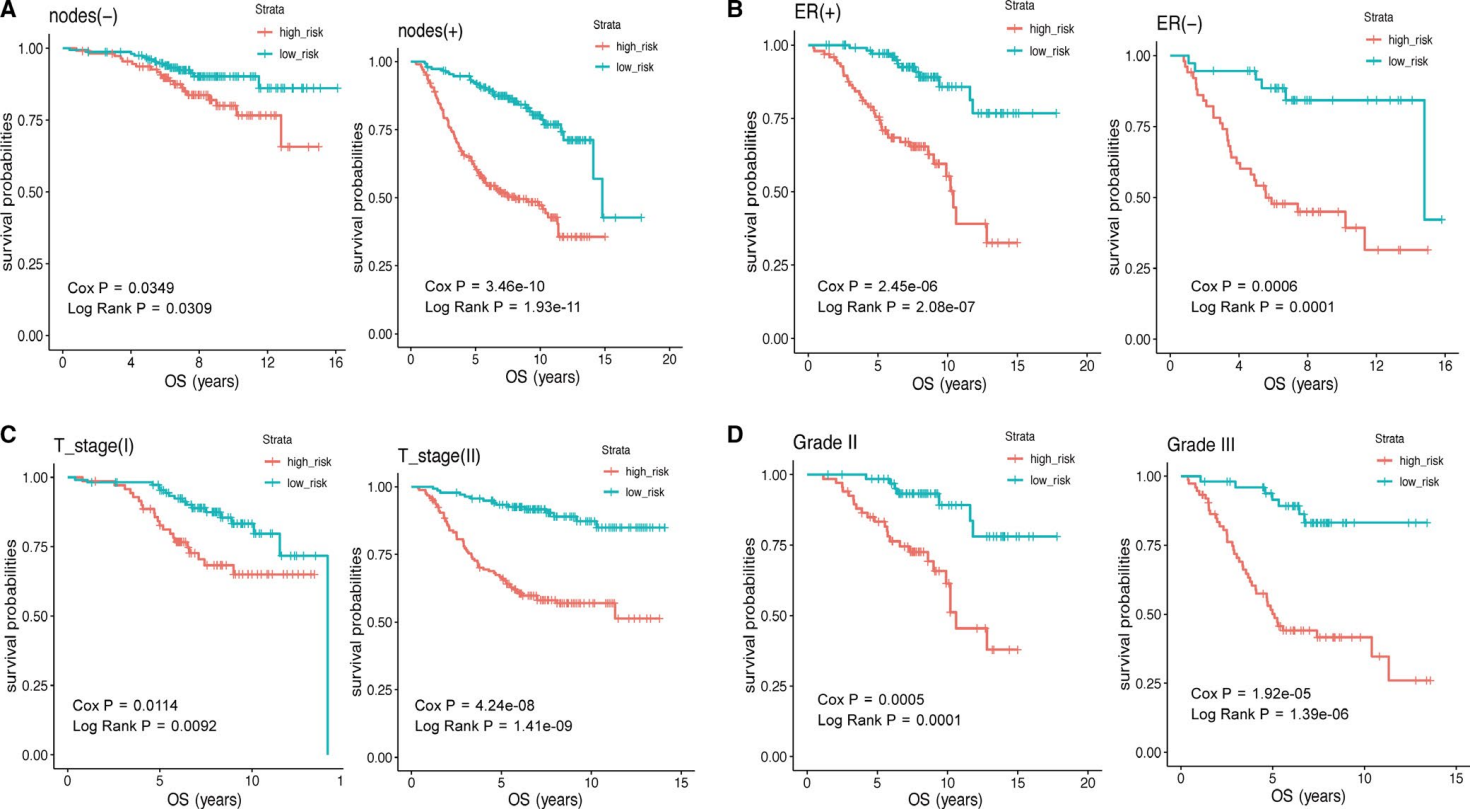
The Kaplan–Meier survival curve was drawn to predict the OS of patients by stratification analysis about nodes (A), ER status (B), T_stage (C), and grade (D). The OS of patients in the low‐risk group was better than that in the high‐risk group. *p* value < 0.05 (log‐rank test) was considered statistically significant

In order to determine whether the 9‐gene signature can be used to predict the OS of patients within the same clinical factor subgroup, we combined the four datasets for stratification analysis. We analyzed the patients in the entire cohort according to the lymph node metastasis status (nodes), ER status, T_stage, grade (due to the number of patients in grade I was too small, only grade II and grade III were analyzed), and divided the patients into high‐risk and low‐risk groups. The results of the Kaplan–Meier plot demonstrated that in the same clinical subgroup, the OS of patients in the low‐risk group is higher than that in the high‐risk group (nodes(‐): HR = 3.72(2.47‐5.60), *p* value = 1.93e‐11; nodes(+): HR = 2.09(1.05‐4.13), *p* value = 3.10e‐02; ER(+): HR = 4.80(2.50‐9.21), *p*_value = 2.08e‐07; ER(‐): HR = 4.73(1.96‐11.44), *p* value = 1.45e‐03; T_stage I: HR = 2.32(1.21‐4.44), *p* value = 9.22e‐03; T_stage II: HR = 4.82(2.75‐8.46), *p* value = 1.41e‐09; Grade II: HR = 4.52(1.94‐10.54), *p* value = 1.32e‐04; Grade III: HR = 5.75(2.58‐12.83), *p* value = 1.39e‐06)(Figure 6).

### 3.6 Relationship between the 9‐gene signature and disease‐free survival

We used DFS data in GSE42568, GSE20711, and GSE88770 datasets to determine the role of the 9‐gene prognostic signature in predicting DFS. The results indicated that the DFS of BRCA patients in the high‐risk group is lower than in the low‐risk group (GSE42568: HR = 3.71 (1.91‐7.21), *p* = 3.28e‐05; GSE20711: HR = 1.82 (0.95‐3.51), *p* = 6.88e‐02; and GSE88770: HR = 2.59 (1.09‐6.17), *p* = 2.59e‐02) (Figure S2A). The time‐varying ROC curve also further verified that the 9‐gene prognostic signature exhibited a substantially effective performance in predicting DFS (Figure S2B).

### 3.7 Validation of 9‐gene prognostic signature

**FIGURE 7:**
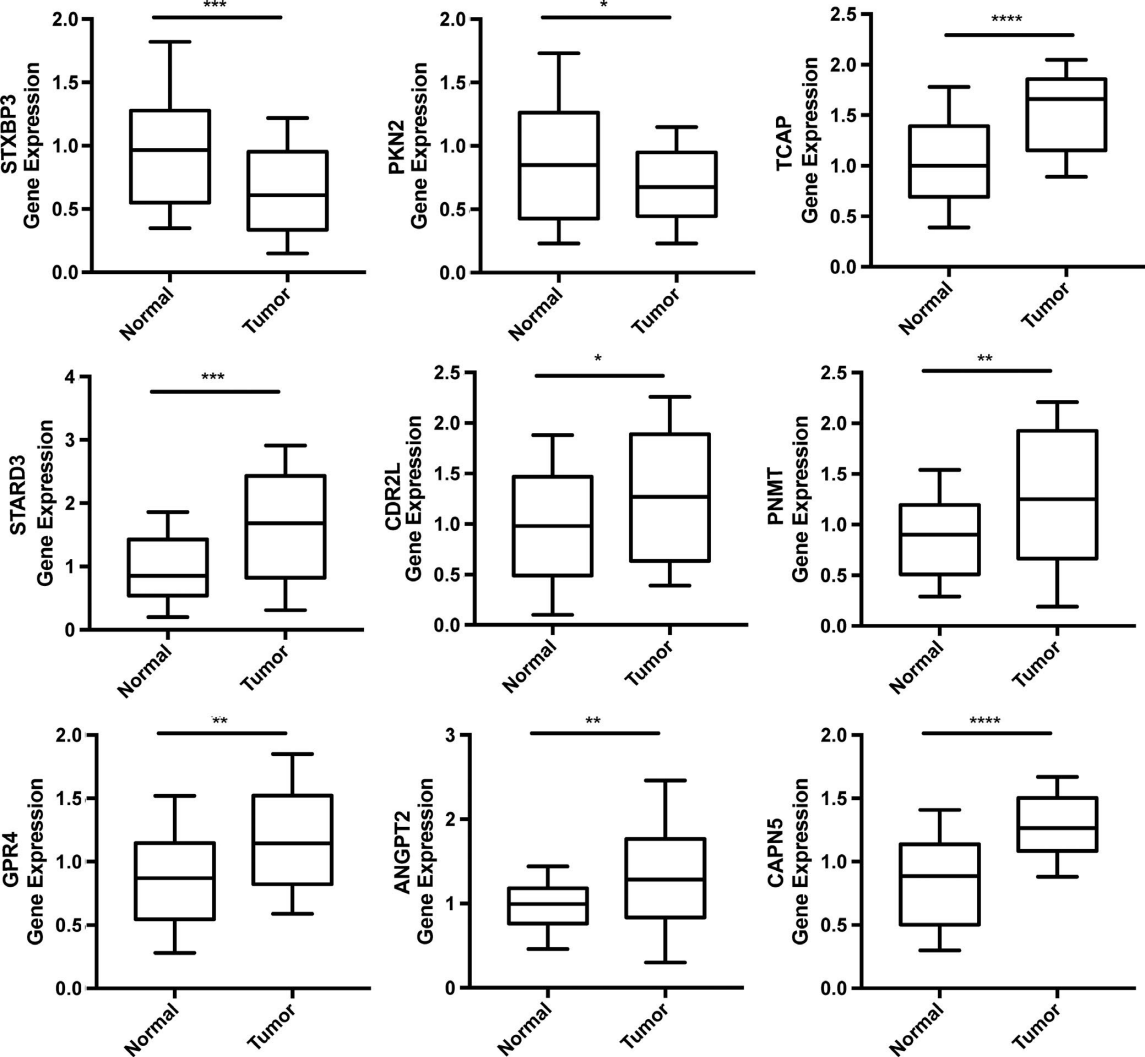
Experimental validation of 9‐gene prognostic signature in BRCA and paracancerous tissues by RT‐PCR. The result indicated that the protective prognostic factors in BRCA patients were significantly downregulated, whereas the dangerous prognostic factors were upregulated. *p* value < 0.05 (*t* test) was considered statistically significant. *p < 0.05; **p < 0.01; ***p < 0.001；****p < 0.0001.

We used the GSE16446, GSE7930, and TCGA‐BRCA datasets for external validation. The KM plot showed that the 9‐gene prognostic signature has good predictive ability, and the ROC working curve also indicated that the gene signature has good working efficiency (Figure S3). To further verify the accuracy of the 9‐gene prognostic signature, we detected the expression levels of *STXBP3*,*PKN2*,*TCAP*,*STARD3*,*CDR2L*,*PNMT*,*GPR4*,*ANGPT2*, and *CAPN5* in BRCA and adjacent tissues by RT‐PCR. A total of 40 pairs of samples were used for analysis. The experimental results revealed that the protective prognostic factors in BRCA patients were significantly downregulated, whereas the dangerous prognostic factors were upregulated (Figure 7).

### 3.8 Gene set enrichment analysis

**FIGURE 8:**
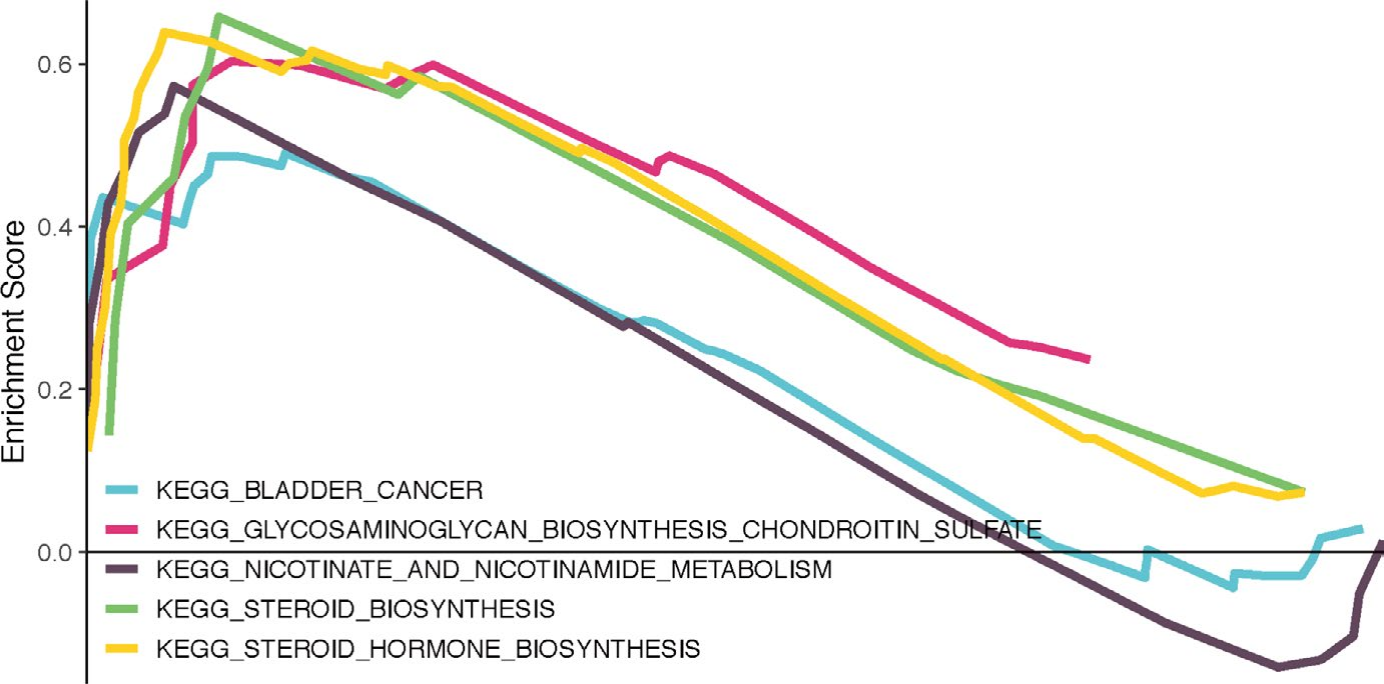
Gene set enrichment analysis

Finally, we used GSEA enrichment analysis to better determine the biological function of the 9‐gene prognostic signature. The top 5 KEGG pathways enriched in high‐risk and low‐risk sample groups were shown according to the FDR <25% cut‐off criteria: bladder cancer, glycosaminoglycan biosynthesis chondroitin sulfate, nicotinate and nicotinamide metabolism, steroid biosynthesis, and steroid hormone biosynthesis (Figure 8).

## 4 DISCUSSION

BRCA is the cancer with the highest incidence in women worldwide.^1^ The comprehensive treatment strategy for BRCA mainly includes surgical resection, chemotherapy, radiation therapy, and targeted therapy.^17^, ^18^, ^19^, ^20^ Effective screening methods will be helpful in reducing the mortality of BRCA.^21^, ^22^ However, the benefits and harms of BRCA screening have been hotly debated in recent years. According to Løberg M's research, the relative number of over‐diagnosis (including ductal carcinoma in situ and invasiveness carcinoma) was 31%.^23^, ^24^ At the same time, the prognosis of advanced BRCA is not optimistic. Even after standardized treatment, many patients eventually develop distant metastases and die from this disease.^18^ New BRCA prognostic markers must be developed to provide guidance and direction for the risk stratification and individualized treatment of BRCA patients.

Syntaxin‐binding protein 3 (STXBP3) is involved in fatty acid‐induced insulin resistance in skeletal muscle cells.^25^ In chronic lymphocytic leukemia, lipoprotein lipase can cooperate with STXBP3 to promote chronic lymphocytic leukemia cells apoptosis.^26^ Protein kinase N2 (PKN2) is a serine / threonine protein kinase associated with PKC. It plays an important role in transcription activation, cell cycle, cell adhesion, and migration.^27^, ^28^ Koh H's research indicated that the C‐terminal region of PKN2 can interact with Akt, inhibit threonine phosphorylation at 308 and 473 site of Akt, and specifically downregulate the activity of Akt protein kinase, blocking the activity of AKT signaling pathway and promoting tumor cell apoptosis.^29^ At the same time, PKN2, as a potential tumor suppressor in colon cancer, can inhibit tumor growth by inhibiting the polarization of tumor‐associated macrophages to M2‐like phenotype.^28^ The teneurin C‐terminal‐associated peptides (TCAP) is encoded by four terminal exons of Tenurin. TCAP1 can be independently transcribed into a soluble peptide and can be combined with Latrophilin to mediate cell adhesion, which is related to neuroendocrine diseases.^30^, ^31^ STARD3 can promote the occurrence and development of gastric cancer via activating the PI3 K / AKT signaling pathway.^32^ Yo antibody can bind endogenous CDR2L, and promote the occurrence of paraneoplastic cerebellar degeneration.^33^ Phenylethanolamine N‐methyltransferase (PNMT) is a rate‐limiting enzyme in adrenaline synthesis and is specifically expressed in adrenergic neurons. PNMT protein at the axon end is reduced in the brains of patients with Alzheimer's disease.^34^ Zhong M's research found that the proton‐sensing receptor GPR4 is highly expressed in colorectal cancer, and GPR4 can promote the metastasis of colorectal cancer cells by inhibiting LATS activity and YAP1 nuclear translocation.^35^ Chen Z's research indicated that DARPP‐32 can further regulate the angiogenic effect of ANGPT2 by inducing STAT3 phosphorylation in gastric tumors.^36^ CAPN5 activation can promote the proteolysis and degradation of a variety of substrates, thereby inducing degeneration of the retina and nervous system.^37^ CAPN5 can also regulate retinal pigment epithelial cell proliferation by regulating SLIT2 cleavage.^38^

Among the top 5 KEGG pathways enriched by GSEA, the pathways associated with tumors are bladder cancer, glycosaminoglycan biosynthesis chondroitin sulfate, steroid biosynthesis, and steroid hormone biosynthesis. Chondroitin sulfate is attached to the core protein to form the chondroitin sulfate proteoglycans.^39^ Chondroitin sulfate proteoglycans accumulated in the matrix of tumor cells, which plays a vital role in promoting the proliferation and invasion of tumor cells by driving multiple oncogenic pathways, such as JNK and tyrosine kinase signaling pathways.^40^, ^41^ Chondroitin sulfate proteoglycans can also promote tumorigenesis by promoting key interactions in tumor microenvironment.^41^ Chondroitin sulfate proteoglycan is up‐regulated in fibrosarcoma, colorectal metastatic cancer, melanoma, and glioma.^40^, ^41^, ^42^, ^43^ To date, the role of chondroitin sulfate proteoglycans in BRCA has not been studied. However, the above‐mentioned mechanisms of chondroitin sulfate proteoglycans in other tumors may provide a reference direction for our future research on BRCA. Moreover, estrogen, as a steroid hormone, plays a vital role in the occurrence and development of BRCA by regulating steroid receptor ER. In addition, ER could crosstalk with other steroid hormone receptors, such as Progesterone receptor, Androgen receptor, and Glucocorticoid receptor, which further affects the development of BRCA.^44^ These 9 genes in our current prognostic model may participate in the occurrence and development of BRCA through the above‐mentioned mechanisms.

In this study, we constructed and validated a 9‐gene prognostic signature (*STXBP3*,*PKN2*,*TCAP*,*STARD3*,*CDR2L*,*PNMT*,*GPR4*,*ANGPT2*,*and CAPN5*) to predict the OS of BRCA patients. We used GSE20685, GSE42568, GSE20711, and GSE88770 datasets for analysis, and finally selected 9 common genes in these four datasets to build a prognostic gene signature. The Kaplan–Meier plot indicated that the OS and DFS of patients in the low‐risk group were higher than those in the high‐risk group. Due to the differences in the quality of expression profiling arrays and the existence of individual differences in patients, the diagnostic efficiency in ROC curves and HR values were differences between cohorts. Nevertheless, in general, the 9‐gene signature showed good diagnostic efficiency for OS events. The nomogram was developed, which combined the 9‐gene prognostic signature and other clinicopathological risk factors, and accurately predicted the 3‐ and 5‐year survival probability of BRCA patients. The calibration plot and C index verification proved that the nomogram has good prediction performance. In addition, the results of multivariate COX regression analysis and stratification analysis revealed that the 9‐gene prognostic signature can exist as an independent risk factor. More convincingly, the RT‐PCR results demonstrated that the mRNA expression of protective prognostic factors in prognostic signature was upregulated in adjacent samples, and dangerous prognostic factors expression presented the opposite results. All of the above results proved that patients can be divided into high‐risk and low‐risk groups successfully through this 9‐gene prognostic signature, which can further be an effective prognostic indicator for BRCA patients.

Several previous studies have reported prognostic signatures for BRCA.^45^, ^46^, ^47^, ^48^, ^49^, ^50^, ^51^, ^52^ Compared with these models, our prognostic model in this article has following advantages. First, this 9‐gene signature showed excellent diagnostic efficiency for both OS and DFS events. In addition, we evaluated the expression levels of 9 genes in the prognostic signature through RT‐PCR experiments, which further validates our bioinformatic results. Nevertheless, this study also has some limitations. Since only external validation sets were used in our analysis, future studies should also set internal validation sets.

Additionally, as BRCA may exhibit different pathogenesis processes and prognosis in diverse subtypes, it could be more accurate and meaningful to develop a prognostic model for distinct BRCA subtypes. Moreover, this prognostic model needs to be verified by further experiments before it is applied to clinic.

## 5 CONCLUSIONS

This study not only constructed a 9‐gene prognostic signature, but also combined with clinical information to further establish a nomogram to better predict the survival probability of patients. In addition, the prediction model does not depend on other clinical case factors. The establishment of this model may help BRCA patients to formulate more accurate treatment plans and improve the prognosis of BRCA.

## CONFLICT OF INTEREST

The authors claim no conflict of interest.

## AUTHOR'S CONTRIBUTIONS

Zelin Tian write this article, Gaosong Wu revise this article. Janing Tang, Xing Liao, Qian Yang, and Yumin Wu conducted all statistical analyses.

## Supporting information

Fig S1

Fig S2

Fig S3

Table S1

Table S2

Table S3

## Data Availability

The data that support the findings of this study are available in TCGA database at https://portal.gdc.cancer.gov/, and in Gene Expression Omnibus databases at https://www.ncbi.nlm.nih.gov/geo/query/acc.cgi, reference number: GSE20685, GSE42568, GSE20711, GSE88770, GSE7390, and GSE16446.
