# Supplementary figures and images for: Identification of a 9‐gene prognostic signature for breast cancer
Source: Cancer Med. 2020 Oct 14;9(24):9471–84. doi: 10.1002/cam4.3523 (PMC7774725; doi:10.1002/cam4.3523)

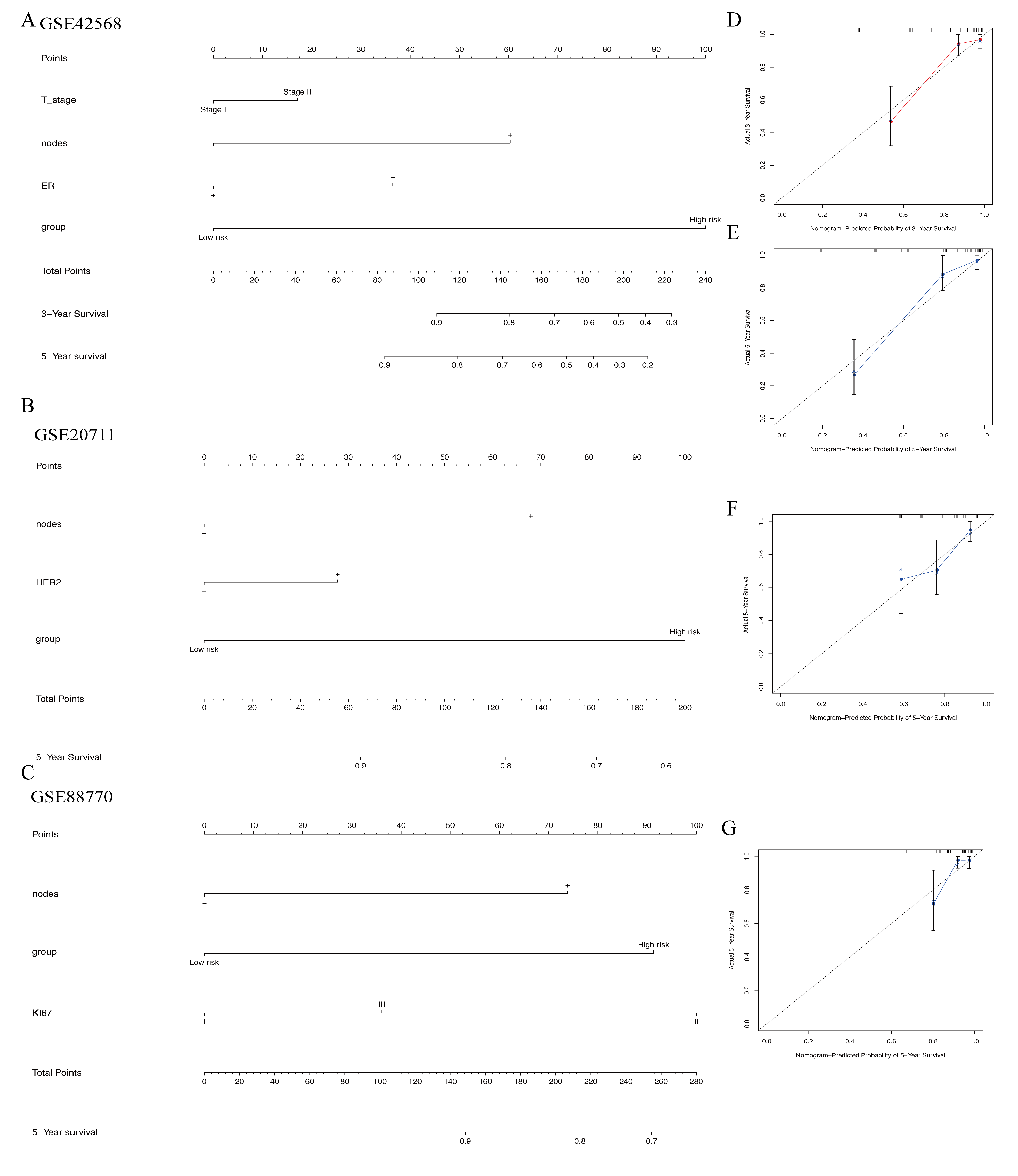

Supplement: Supplementary file 1 — Fig S1 [file CAM4-9-9471-s001.tif]

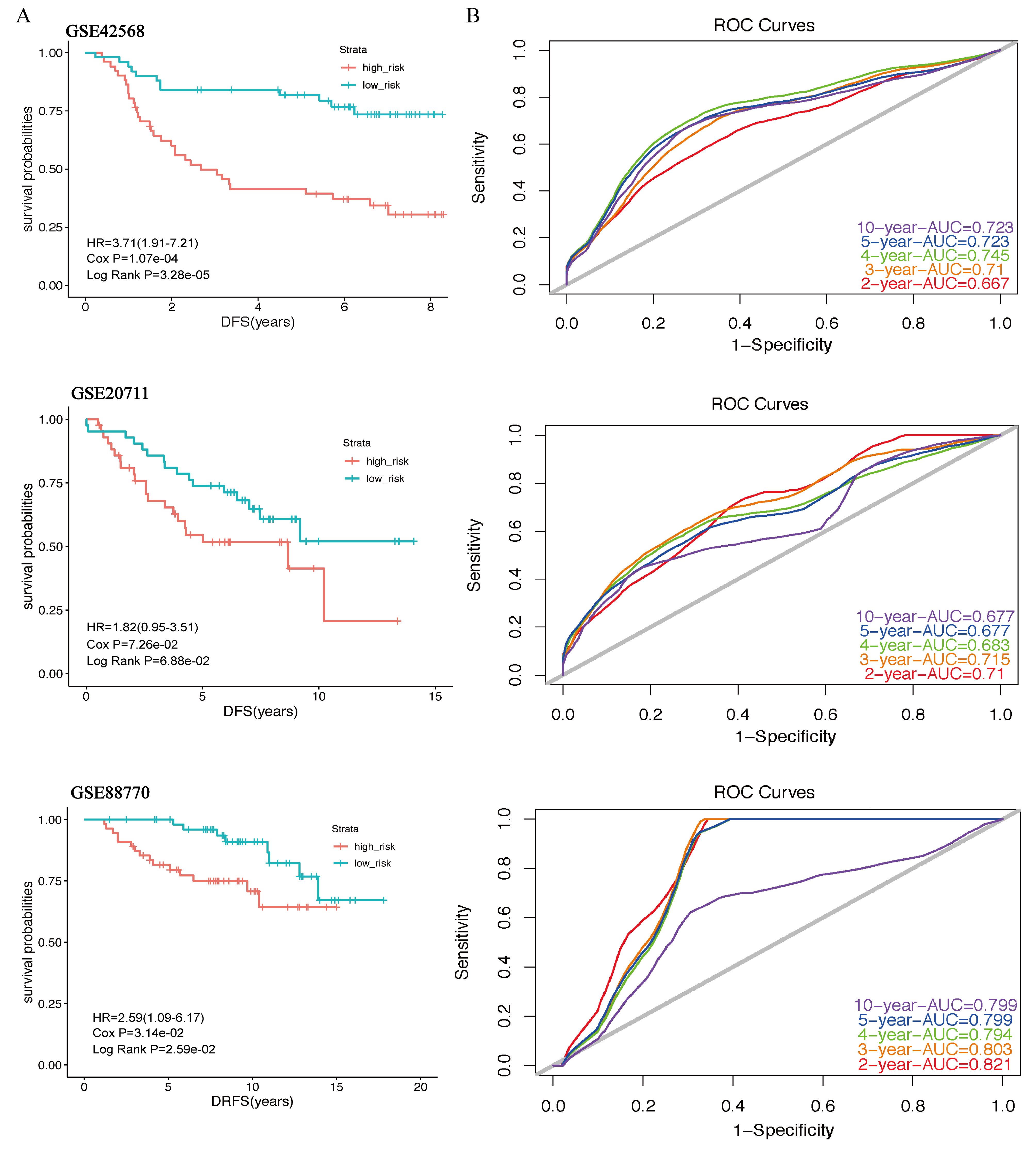

Supplement: Supplementary file 2 — Fig S2 [file CAM4-9-9471-s002.tif]

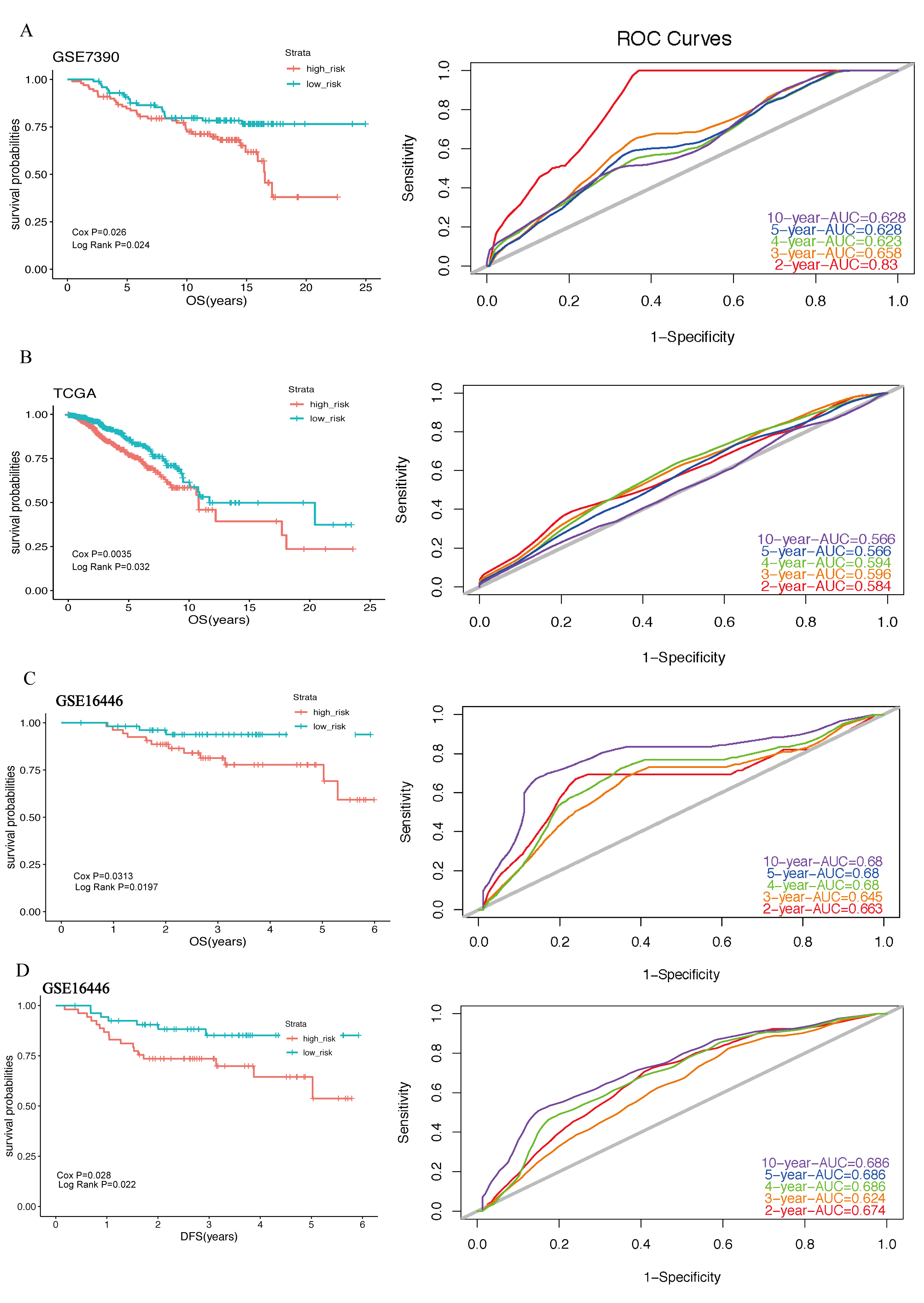

Supplement: Supplementary file 3 — Fig S3 [file CAM4-9-9471-s003.tif]
