# Supplementary material for: Identification of a 9‐gene prognostic signature for breast cancer
Source: Cancer Med. 2020 Oct 14;9(24):9471–84. doi: 10.1002/cam4.3523 (PMC7774725; doi:10.1002/cam4.3523)
Supplement: Supplementary file 4 — Table S1 [file CAM4-9-9471-s004.docx]

| **Clinical features and molecular characteristics (cases)** | | | | |
| --- | --- | --- | --- | --- |
| **ERα** |  | **+** | 25 |  |
|  |  | **-** | 15 |  |
| **PR** |  | **+** | 21 |  |
|  |  | **-** | 19 |  |
| **HER2** |  | **+** | 22  18 |  |
|  |  | **-** |  |  |
| **Lymph node metastasis** |  | **+** | 20  20 |  |
|  |  | **-** |  |  |
| **Pathological grade** |  | Low | 1  24  15 |  |
|  |  | Medium |  |  |
|  |  | High |  |  |
