# Supplementary material for: Identification of a 9‐gene prognostic signature for breast cancer
Source: Cancer Med. 2020 Oct 14;9(24):9471–84. doi: 10.1002/cam4.3523 (PMC7774725; doi:10.1002/cam4.3523)
Supplement: Supplementary file 5 — Table S2 [file CAM4-9-9471-s005.docx]

| Target Gene | Primer Sequence |
| --- | --- |
| STXBP3 |  |
| forward | 5′- AGCAACAGTGTTTGATGACTGC-3′ |
| reverse | 5′- TCTGACAGGTTCACGGTTCTTA-3′ |
| PKN2 |  |
| forward | 5′- TGACCCTCGTTGTTCTACTAGC-3′ |
| reverse | 5′- GTTTCCGATCCTTTGAAGATCCA-3′ |
| TCAP |  |
| forward | 5′- GTCGGAGGAGAACTGTGAGC-3′ |
| reverse | 5′- ATGCCCATCCGCATCATCAG-3′ |
| STARD3 |  |
| forward | 5′- TACCAACACAGGCATCCGTAA-3′ |
| reverse | 5′- GCCAGGACAAAGATGTCGAAG-3′ |
| CDR2L |  |
| forward | 5′- TGGGCTGACGGAGACCATT-3′ |
| reverse | 5′- TGTAGGCGGAAAGCATCCTTG-3′ |
| PNMT |  |
| forward | 5′- CGCACCCTCATCGACATTG-3′ |
| reverse | 5′- AGCTGGCGCTCCTTATCCT-3′ |
| GPR4 |  |
| forward | 5′- CTCTTCCGAGACCGCTACAAC-3′ |
| reverse | 5′- ACACCCGATAGAGGTTCATCC-3′ |
| ANGPT2 |  |
| forward | 5′- AACTTTCGGAAGAGCATGGAC -3′ |
| reverse | 5′- CGAGTCATCGTATTCGAGCGG -3′ |
| CAPN5 |  |
| forward | 5′- CGCCACTGACGACTCACTC-3′ |
| reverse | 5′- CTGCCACAAACCAGCAGTTG-3′ |
| GAPDH |  |
| forward | 5′-GAAGGTGAAGGTCGGAGTC-3′ |
| reverse | 5′-GAAGATGGTGATGGGATTTC-3′ |
