# Supplementary material for: Identification of a 9‐gene prognostic signature for breast cancer
Source: Cancer Med. 2020 Oct 14;9(24):9471–84. doi: 10.1002/cam4.3523 (PMC7774725; doi:10.1002/cam4.3523)
Supplement: Supplementary file 6 — Table S3 [file CAM4-9-9471-s006.docx]

| **Gene name** | **Gene type** | **Gene type** | **Location** |
| --- | --- | --- | --- |
| TXBP3 | Protective prognostic factor | Protein Coding | Chromosome 3, NC_000069.6 |
| PKN2 | Protective prognostic factor | Protein Coding | Chromosome 1, NC_000001.11 |
| TCAP | Dangerous prognostic factor | Protein Coding | Chromosome 17, NC_000017.11 |
| STARD3 | Dangerous prognostic factor | Protein Coding | Chromosome 17, NC_000017.11 |
| CDR2L | Dangerous prognostic factor | Protein Coding | Chromosome 17, NC_000017.11 |
| PNMT | Dangerous prognostic factor | Protein Coding | Chromosome 10, NC_005109.4 |
| GPR4 | Dangerous prognostic factor | Protein Coding | Chromosome 19, NC_000019.10 |
| ANGPT2 | Dangerous prognostic factor | Protein Coding | Chromosome 8, NC_000008.11 |
| CAPN5 | Dangerous prognostic factor | Protein Coding | Chromosome11, NC_000011.10 |
